# One-year longitudinal study of the stratum corneum proteome of retinol and all-*trans*-retinoic acid treated human skin: an orchestrated molecular event

**DOI:** 10.1038/s41598-023-37750-5

**Published:** 2023-07-11

**Authors:** Kahina Abed, Aude Foucher, Dominique Bernard, Emmanuelle Tancrède-Bohin, Nükhet Cavusoglu

**Affiliations:** 1 L’Oréal Research and Innovation, Aulnay-Sous-Bois, France; 2 Service de Dermatologie, Hôpital Saint-Louis, Paris, France

**Keywords:** Mass spectrometry, Proteomic analysis, Biomarkers, Proteomics

## Abstract

Topically applied all-*trans-*retinoic acid (RA) is a gold-standard anti-aging molecule used in dermatology. As its cosmetic counterpart used in anti-aging, Retinol (ROL) is also a known metabolic precursor of RA. Despite this metabolic link, they haven’t been compared exhaustively in vivo at a mechanistic level. Therefore, to highlight the effect of a topical application of both molecules on in vivo skin, we undertook a longitudinal 1-year study and performed an untargeted proteomic analysis to get a more holistic view on the underlying biological mechanisms of action. The generation of the temporal proteomics signatures of retinol and all-*trans*-retinoic acid reveals the impact of these molecules on biological functions related to the aging of skin. New biological functions impacted by retinoids were discovered: glycan metabolism and protein biosynthesis. In addition, the temporal analysis reveals highest modulations at early time points while the physical measures, such as epidermal thickening, was mostly observed at the latest time point, demonstrating a strong time lapse between molecular and morphological impacts. Finally, these global temporal signatures could be used to identify new cosmetic compounds of interest.

## Introduction

Skin aging is a complex process that is a consequence of genetic and environmental interactions. While chronological aging is relentless, the biological and physical results of chronological aging vary in their kinetics, biological events, and clinical manifestations.

Since clinical changes in skin are among the most visible signs of aging, skin appearance has a significant emotional and psychological impact on our quality of life. As well as appearance, skin aging has a significant impact on age-related skin diseases, notably impaired wound healing in the elderly suggesting a dysregulation of molecular mechanisms during the aging process^1–4^. In particular, skin proteome has been shown to be altered during aging^5^.

Topical All-*trans*-retinoic acid (RA), used in dermatology for its benefits in the treatment of acne as well as for various keratinization and pigmentation disorders, is the gold-standard treatment prescribed by dermatologists for treating photodamaged skin^6–8^. Topical retinol (ROL), its precursor, has also been used for decades in topical anti-aging cosmetics^9^. While their impact on aging’s clinical, immuno-histochemical or molecular parameters have been extensively studied, the molecular mechanisms of action of ROL and RA in a temporal manner remains unexplored.

To fill this knowledge gap, we followed the molecular events taking place in the *stratum corneum* (SC) after topical application of ROL and RA over the course of 1 year. We used a state-of-the-art proteomic approach, combining liquid chromatography-tandem mass spectrometry (LC–MS/MS) and isobaric markers for relative quantification, to produce to our knowledge the first in vivo temporal signature of ROL and RA topical application on in vivo skin.

## Materials and methods

### Clinical study

The clinical study was conducted in Paris, France, between February 2011 and April 2012, and the experimental protocol was approved by the Saint Louis Hospital ethics committee (EC reference 2010/58), complying with the Declaration of Helsinki and all volunteers gave written informed consent. Clinical and epidermal changes including morphology, melanin content and distribution assessed by histology and in vivo multiphoton microscopy have already been published^10^.

This study involved 30 female volunteers (50–65y) with constitutive skin color determined by ITA value between 10° and 41° (ITA group III/IV). They applied Retinol 0.3% (ROL 0.3% cream, L’Oréal group) (n = 15) or all-*trans*-Retinoic acid 0.025% (RA 0.025% cream, Galderma) (n = 15) on one dorsal forearm versus a control product (white paraffin containing excipient, Bayer) on the other forearm for 1 year.

**Figure 1:**
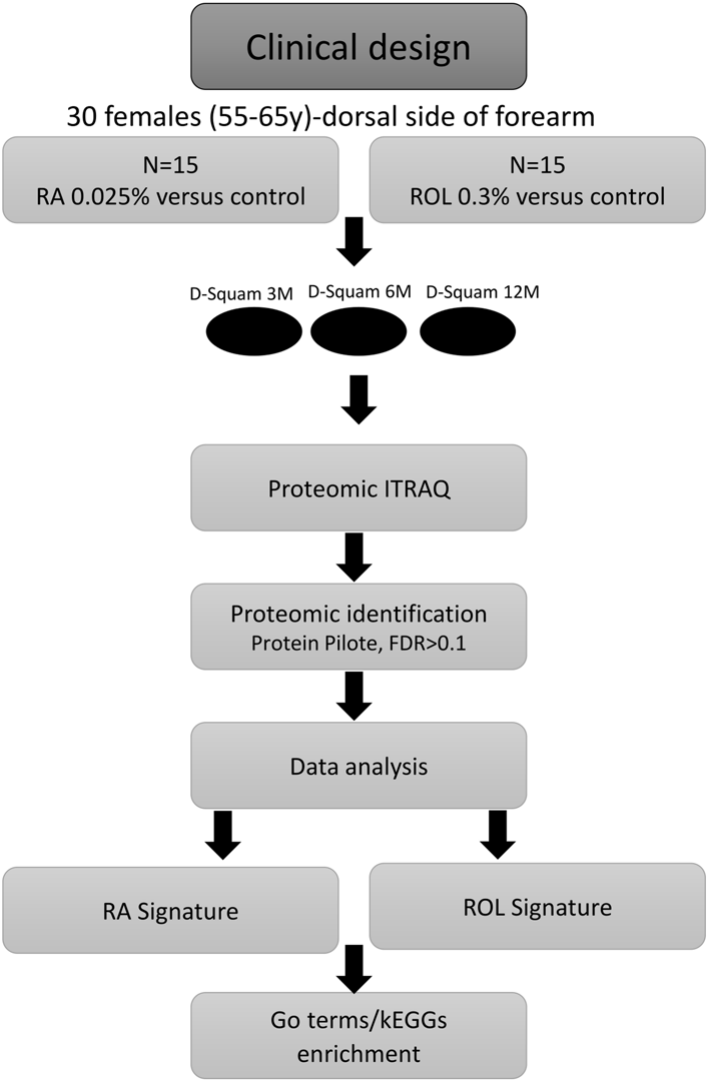
Scheme of overall Clinical and analytical design of the study. The clinical study was carried out on a total of 30 subjects, 15 of whom received a daily application of RA at 0.025% and 15 others of ROL at 0.3%. Samplings were carried out at 3 months (3 M), 6 months (6 M) and 12 months (12 M). Protein extractions were performed from everyone from D-squams®. Each iTRAQ (isobaric Tags for Relative and Absolute Quantitation) experiment allowed the comparison of a group treated with RA against the same group control, and a group treated with ROL against the same group control.

Non-invasive strippings (D-squams®) were performed on the treated and non-treated arms at 3 months (June), 6 months (September), and 12 months (March + 1 yr), for each volunteer. These groups were further divided in three sub-groups for ROL treated and 3 sub-groups for the ROL control group before iTRAQ labeling of each sub-group, thus giving a triplex experiment of each condition. The same design was applied for the RA treatment (Fig. 1).

Areas of investigation were identified by tracing on transparent plastic sheets (Monaderm, Monaco) natural anatomic marks (external and internal forearm edges, ulna head, elbow fold) and also skin folds and neavus on volunteers in a standardized position.

### Protein extraction, iTRAQ labeling and mass spectrometry analysis

Protein extractions were performed from each individual from D-squams® strippings as published earlier^11^. Briefly, proteins were extracted in a deoxycholate buffer using mechanical lysis. After precipitation in 4 volumes acetone, proteins were resolubilized, reduced by TCEP and alkylated using methyl methanethiosulfonate (MMTS) prior to digestion by trypsin.

Tryptic peptides were labeled using iTRAQ 4 plex reagent before desalting. Fractionation of the peptides was performed in gel using isoelectric focusing on a 3–10 pH gradient gel strip to generate *in fine* 18 fractions. Each fraction was analyzed by mass spectrometry on a 5600 Triple-TOF (ABSciex, Framingham, USA) coupled to an HPLC (Agilent 1200). The experimental design permitted three independent LC–MS/MS analyses. Briefly 500 ng of samples were injected onto a trapping column for desalting followed by a chromatographic separation (nanoLC) on a 75 µm ID x 15 cm with particle size of 5µm Reverse Phase (RP) C18 column. Samples were run using a 90 min gradient from 10 to 35% solvent B (solvent A being a solution of 0.1% formic acid and solvent B: 0.1% formic acid in acetonitrile) at a flow rate of 300 nl/min. Data were acquired using a positive ESI mode with a voltage of 2.4 kV in positive mode. Full scans were obtained in the mass range of 400–1250 in 250 ms. Data dependent acquisition (DDA) was used for data collection for the high intensity ions in the m/z range 100–1600.

### Protein identification

Data files were submitted for simultaneous searches using Protein Pilot version 4.0 software (AB Sciex) and Mascot search engine (Matrix Science) with the following criteria: Trypsin digestion, fixed modification on cysteine of methylthio, variable modification iTRAQ (4 plex iTRAQ (K), iTRAQ (N-term), iTRAQ (Y)) and methionine oxidation. Peptide charge was restricted to +2, +3 and +4. Peptide mass tolerance restricted to or set at (± 0.1) and fragment mass tolerance was ± 0.1. Proteins were defined as differentially modulated if they fit the following criteria: at least two peptides with high confidence (95%) for identification (*p*-value < 0.05) and with a fold change of proteins greater or equal to |1.5|.

### ELISA

Sandwich ELISA assay was performed to quantify transglutaminase 3 (TGM3) using an in-house developed assay using Meso Scale technology (Meso Scale Discovery). Custom made HuCal (human combinatorial antibody library) antibodies were developed by AbD Serotec BioRad for these ELISAs. For TGM3 ELISA, standard curve was produced using recombinant TGM3 (Zedira, #T024), capture antibody (HuCal_AbD12765) and detection antibody (HuCal_AbD12866). An 8 points standard curve (0–0.5 mg/ml (0.0075, 0.0156, 0.0313, 0.0625, 0.125, 0.25, 0.5) was made in triplicate. Samples were diluted 1/5 in MSD sample diluent and 25 µl was loaded per well. Detection was performed using anti-species sulfo-tagged antibodies from Meso Scale Discovery. Plates were read in a MesoSector S6000 plate reader (Meso Scale Discovery).

### Data analysis

Differentially expressed proteins of retinoids treated subject and vehicle subject, were defined by a fold change ≥|1.5| in 2 or 3 replicates. The biological interpretation of these protein lists was performed by enrichment calculations using the R ClusterProfiler package^12^ and by using the "Gene Ontology" databases^13^. A term from Gene Ontology or a Pathway from the KEGG database was significantly impacted when it had a corrected *p*-value < 0.05 with the list of proteins given as input^14^. Heatmap or volcano plot type visualizations were also generated with the R tool to facilitate visualization and biological interpretation of the results.

## Results

To investigate the molecular mechanisms by which topical application of ROL or RA acts on the skin, we used an iTRAQ based proteomics approach to highlight all the proteins and therefore functions involved in those mechanisms. ROL and RA were applied daily on the dorsal photodamaged forearm over 1 year and samples were taken at 3, 6 and 12 months.

**Figure 2:**
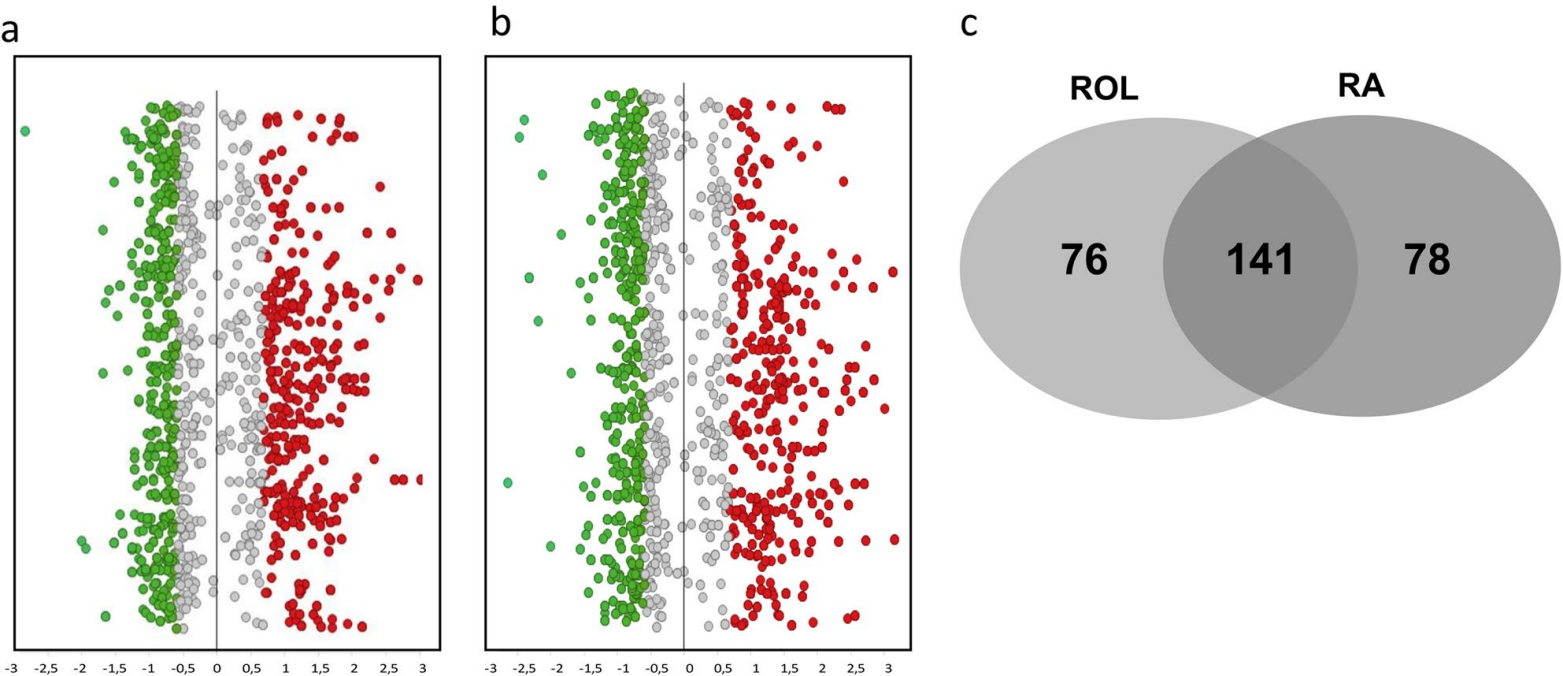
Visualization of DEPs after ROL (**a**) and RA (**b**) treatment. Distribution of protein based on log2-transformed fold change. Red dots indicate upregulated proteins, green dots indicate downregulated proteins and grey dots represents not modulated proteins. (**c**) Venn diagram representation of the differentially modulated proteins by ROL and RA treatment.

Global analysis of differentially expressed proteins (DEPs) revealed a high impact of ROL and RA application on skin’s surface proteome. In summary, 217 proteins (106 upregulated and 111 downregulated) (Fig. 2a) and 219 (106 upregulated and 113 downregulated) (Fig. 2b) were modulated by ROL and RA, respectively. 141 DEPs were modulated by the topical application of both retinoids (65%) and defined the biological signature of each of the retinoids (Fig. 2c).

As a preliminary validation, we focused on two well-known characterized binding proteins shown to bind RA and deliver it to the receptors, FABP5 (Fatty Acid Binding Protein 5) and CRABP2 (Cellular Retinoic Acid Binding Protein 2)^15,16^. Both were upregulated in this study with a fold change up to 8.

**Figure 3:**
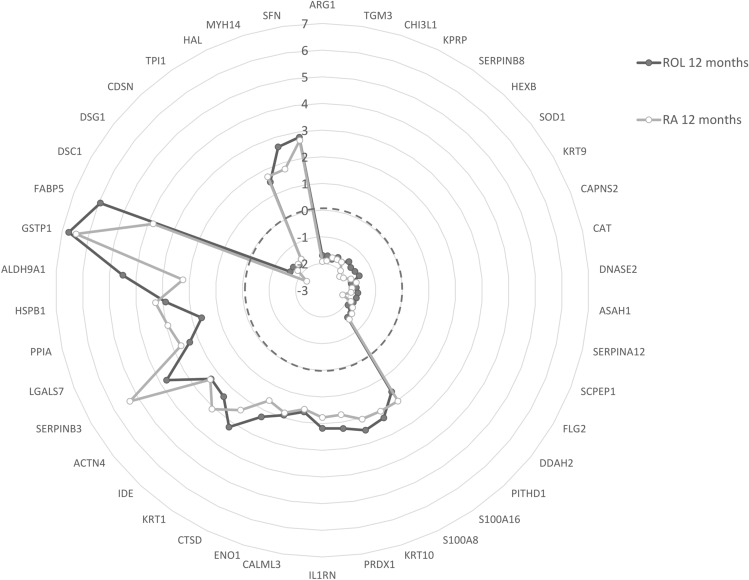
Radar plot representation of the 42 DEPs common to ROL (Black, filled circles) and RA treatments (grey, empty circles) at 12 months. Proteins are identified with their Gene Names. The axis indicates the fold change, while the dotted circle delimitates the up- and downregulated proteins.

Macroscopic analysis of retinoids signatures revealed similar biological impacts in terms of protein levels and their direction of modulation. This is exemplified in the radar plot, showing the 42 DEPS common to ROL and RA at 12 months (Fig. 3).

### Similar global biological impact of ROL and RA

**Figure 4:**
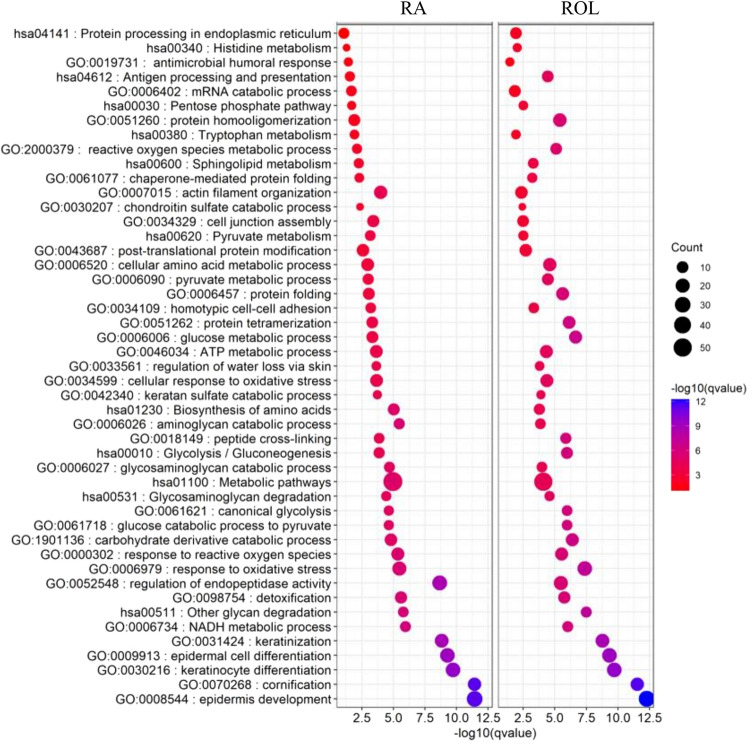
Dotplot representation of selected enriched biological processes and KEGG pathways for RA (left panel) and ROL (right panel). The size of the dots represents the number of proteins implicated in the enrichment. The color code of the dots represents the adjusted *p*-values of the enrichment in − log10 (qvalue).

To elucidate the biological processes and metabolic pathways that are affected by the application of ROL and RA, we performed a Gene Ontology (GO) term and KEGG pathway enrichment analysis on the signatures obtained from the combined time points 3, 6 and 12 months. As expected, no specific biological processes to ROL or RA have been identified (Fig. 4).

To create a meaningful analysis, we condensed the numerous biological processes into 7 superpathways (Table 1) which covered: energy/canonical glycolysis, antimicrobial response, anti-oxidative defense, epidermal barrier-associated proteins, glycan metabolism, cytoskeletal rearrangement, and protein assembly/folding.

**Table 1:** List of the 7 superpathways created from selected biological processes and KEGG pathways.

Superpathways	Selected enriched go and kegg terms
Energy/canonical glycolysis	GO:0061621/canonical glycolysis
	GO:0061718/glucose catabolic process to pyruvate
	GO:1901136/carbohydrate derivative catabolic process
	GO:0006006/glucose metabolic process
	GO:0046034/ATP metabolic process
	GO:0006090/pyruvate metabolic process
	GO:0006734/NADH metabolic process
	hsa00010/Glycolysis
	hsa00620/Pyruvate metabolism
	hsa00030/Pentose phosphate pathway
Antimicrobial response	GO:0019730: antimicrobial humoral response
Anti-oxydative defense	GO:0034599/cellular response to oxidative stress
	GO:2000379/ reactive oxygen species metabolic process
	GO:0000302/response to reactive oxygen species
	GO:0098754/detoxification
	GO:0006979/response to oxidative stress
Epidermal barrier-associated protein	GO:0008544/epidermis development
	GO:0033561/regulation of water loss via skin
	GO:0052548/regulation of endopeptidase activity
	GO:0018149/peptide cross-linking
	GO:0031424/keratinization
	GO:0009913/epidermal cell differentiation
	GO:0030216/keratinocyte differentiation
	GO:0070268/cornification
	hsa00600/Sphingolipid metabolism
Glycan metabolism	GO:0006027/glycosaminoglycan catabolic process
	GO:0030207/chondroitin sulfate catabolic process
	GO:0006026/aminoglycan catabolic process
	GO:0042340/keratan sulfate catabolic process
	hsa00511/Other glycan degradation
	hsa00531/Glycosaminoglycan degradation
	hsa01100/Metabolic pathways
Cytoskeletal rearrangement	GO:0007015/actin filament organization
	GO:0034329/cell junction assembly
	GO:0034109/homotypic cell–cell adhesion
Protein assembly/folding	GO:0006402/mRNA catabolic process
	GO:0043687/post-translational protein modification
	GO:0061077/chaperone-mediated protein folding
	GO:0006520/cellular amino acid metabolic process
	GO:0051260/protein homooligomerization
	GO:0006457/protein folding
	GO:0051262/protein tetramerization
	hsa04612/Antigen processing and presentation
	hsa01230/Biosynthesis of amino acids
	hsa00340/Histidine metabolism
	hsa00380/Tryptophan metabolism
	hsa04141/Protein processing in endoplasmic reticulum

These 7 superpathways were then utilized to evaluate the biological impacts of the retinoids on the skin’s biology and the adaptability of the SC to their application through the temporal signatures. Combining the biological functions with the temporal signature, we were able to define the orchestrated molecular events detected in the SC which reflected what was occurring in the epidermis.

### Temporal evolution of ROL and RA proteomic signatures

**Figure 5:**
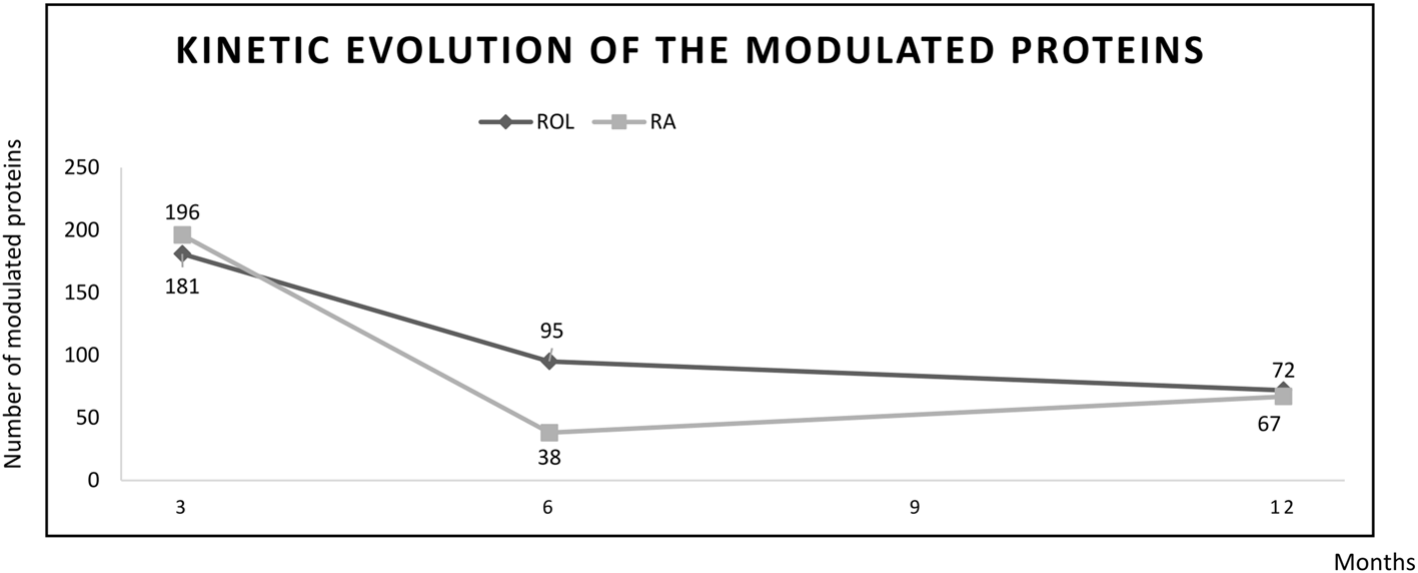
Evolution of the number of modulated proteins over time (months) for ROL (black) and RA (grey).

Temporal signatures were obtained by plotting the number of DEPs at each time point of the analysis. This visualization shows the rapid decline of DEPs impacted by ROL and RA treatment (Fig. 5). To our knowledge, this in vivo temporal signature demonstrates for the first time the evolution in the biological skin response to retinoids treatment.

**Figure 6:**
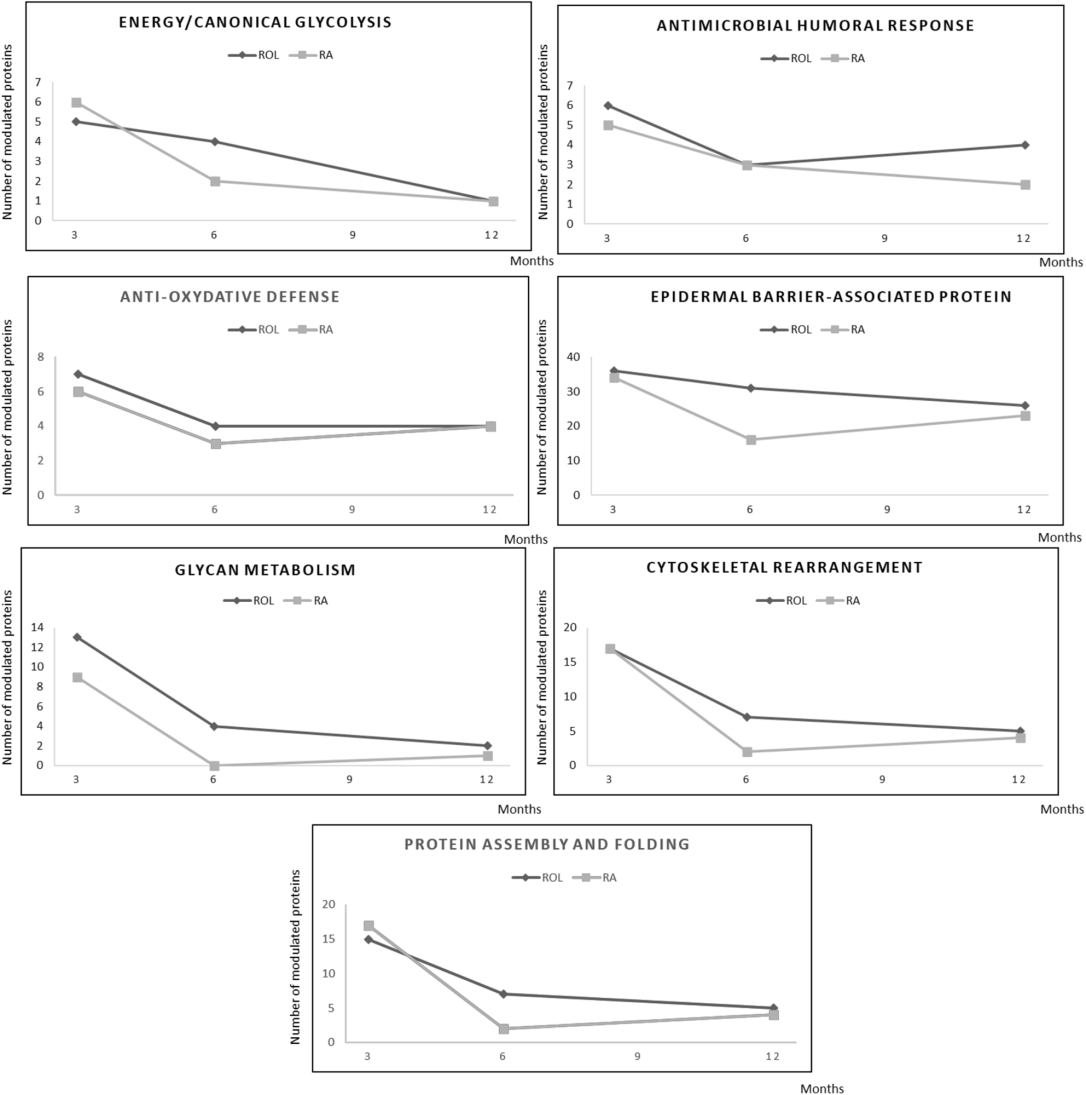
Evolution of the number of DEPs in the 7 superpathways for ROL (black) and RA (grey).

To determine if this global trend was similar for all the biological functions, we analyzed the evolution of each superpathway independently (Fig. 6).

The evolution curves can be classified in three profiles: 1- decreasing temporal signature containing up and down DEPs (i.e.: antimicrobial response, anti-oxidative defense), 2- decreasing temporal signature containing proteins with a similar modulation (energy/canonical glycolysis, cytoskeletal rearrangement, and protein assembly/folding), 3- pathway with stable temporal effect (epidermal barrier-associated protein). For interpretation of the biological impact, we have focused on profiles 2 and 3.

### Early increase of energy production, protein assembly/folding and cytoskeletal rearrangement by ROL and RA

#### Energy/canonical glycolysis

**Figure 7:**
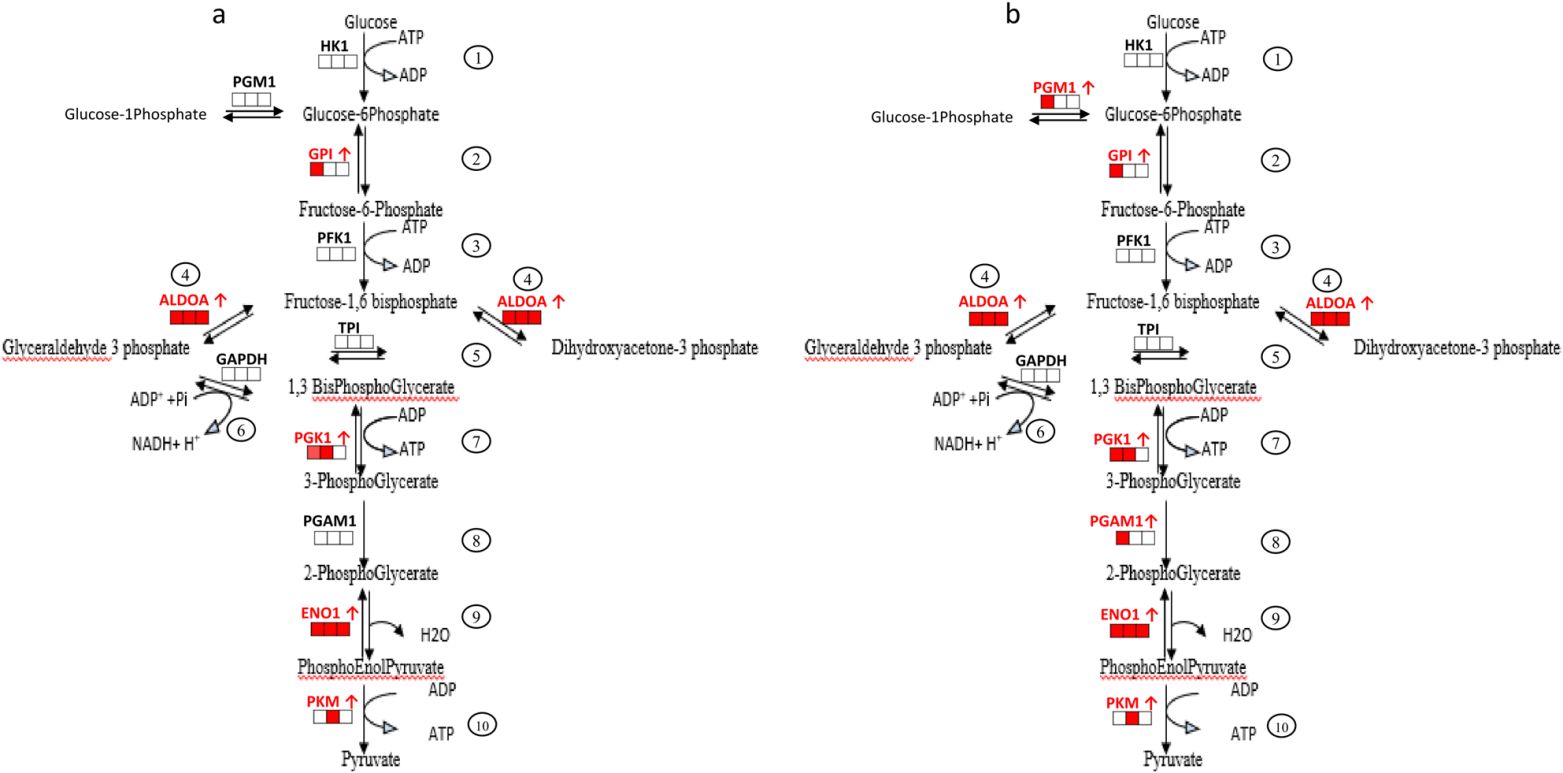
Impact of ROL (**a**) and RA (**b**) application on enzymes of the glycolysis metabolic pathways at 3, 6, and 12 months. Each square represents the up- (red) or non- modulation (white) of the enzymes at 3 (left square), 6 (middle square) and 12 (right square) months.

**Figure 8:**
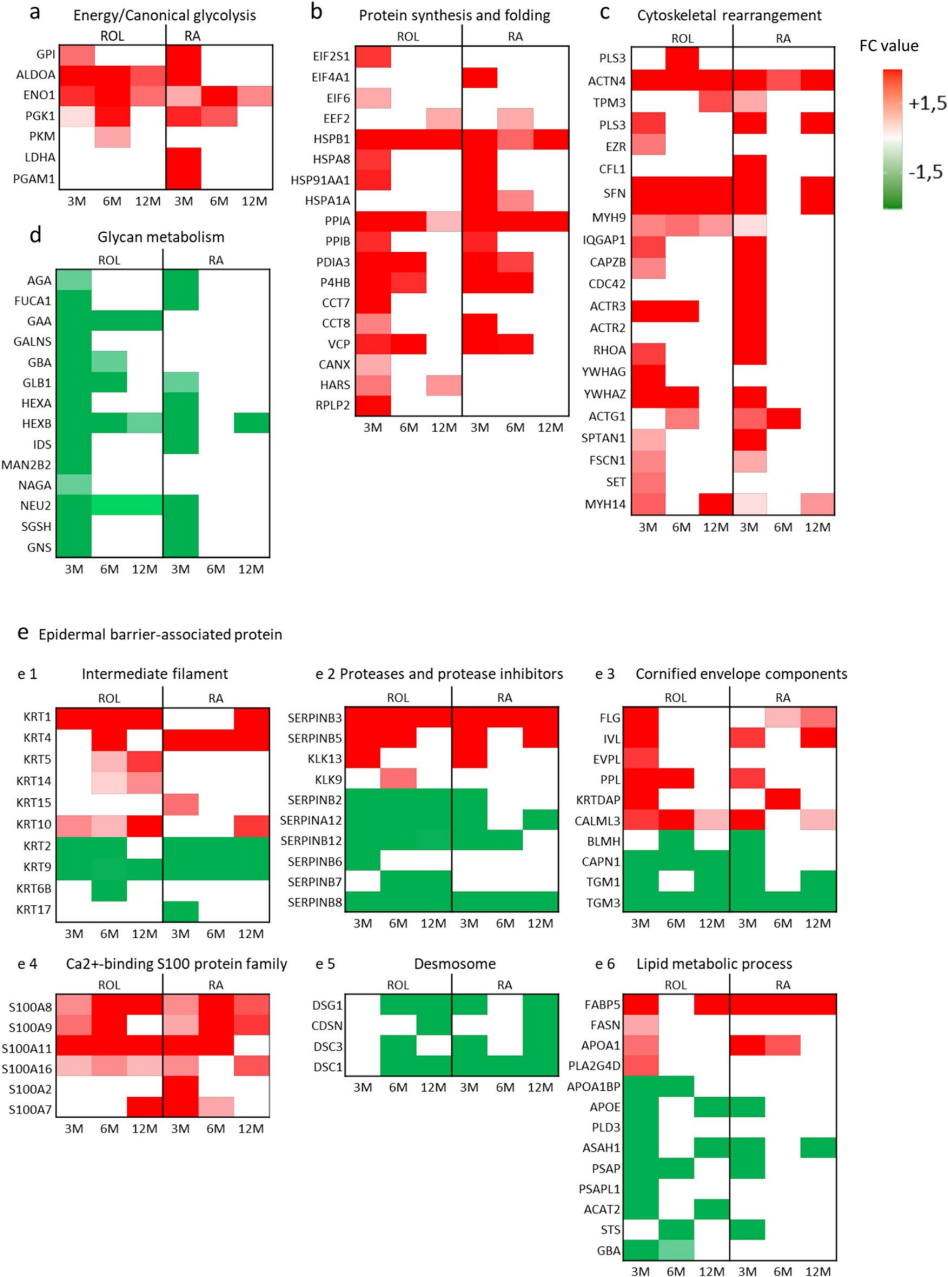
Heatmap of fold-change values of DEPs during ROL and RA treatment for each superpathway. (**a**) Energy/canonical glycolysis, (**b**) Protein assembly/folding, (**c**) Cytoskeletal rearrangement, (**d**) Glycan metabolism (**e**) Epidermal barrier-associated protein: (e1) Intermediate filament, (e2) Proteases and protease inhibitors, (e3) Cornified envelope components, (e3) Ca2 + -binding S100 protein family, (e4) Desmosome, and (e5) Lipid metabolic process. Horizontal axis: time points 3 months (3 M), 6 months (6 M) and 12 months (12 M). Color key: Green denotes downregulation while red denotes upregulation.

The glycolysis pathway exhibited significant modulation by retinoids with six enzymes upregulated and one enzyme downregulated, among the 10 enzymes comprising this pathway. At 3 months of application GPI (Glucose-6-Phosphate-isomerase; FC ROL = 1.9, RA = 2.8), ALDOA (Fructose bisphosphate aldolase A FC ROL = 2.4, RA = 2.4), PGK1 (phosphoglycerate kinase 1 FC ROL = 2.2, RA = 2.2), PGAM1 (phosphoglycerate mutase 1 FC RA = 2.4), ENO1 (enolase FC ROL = 2.1, RA = 1.6), and Phosphoglycerate kinase 1 (PGK1 FC RA = 2.1) were upregulated. Interestingly the early time points 3 and 6 months showed the most important increase in these enzymes (Figs. 7 and 8a).

#### Protein assembly/folding

The protein synthesis/folding was enhanced after both treatments with16 upregulated proteins for ROL and 11 upregulated proteins for RA. The orchestrated molecular events can be defined by the early increase of translation initiation factors EIF2S1 (Eukaryotic translation initiation factor 2 subunit 1), EIF6 (Eukaryotic translation initiation factor 6) and EIF4A1 (Eukaryotic initiation factor 4A-I) at 3 months followed by the subsequent increase in the elongation process factor EEF2 (Eukaryotic translation elongation factor 2) at 6 month (RA) and 12 months (ROL) (Fig. 8b).

#### Cytoskeletal rearrangement

Twenty-one proteins found upregulated by ROL and RA were involved in cytoskeletal rearrangement (Fig. 8c) (18/21 by ROL and 17/21 by RA), with the greatest DEPs observed at 3 months. Among them we identified three protein families, the actinin family proteins ACTN4 (Actinin Alpha 4) and ACTG1 Actin Gamma 1), the myosin superfamily proteins MYH9 (Myosin-9) and MYH14 (Myosin-14), and the 14-3-3 family proteins YWHAG (14-3-3 protein gamma) and YWHAZ (14-3-3 protein zeta/delta). Once again, the protein levels exhibited the most important increases at the early time points of 3 and 6 months.

### Decreased glycan metabolism by ROL and RA

**Figure 9:**
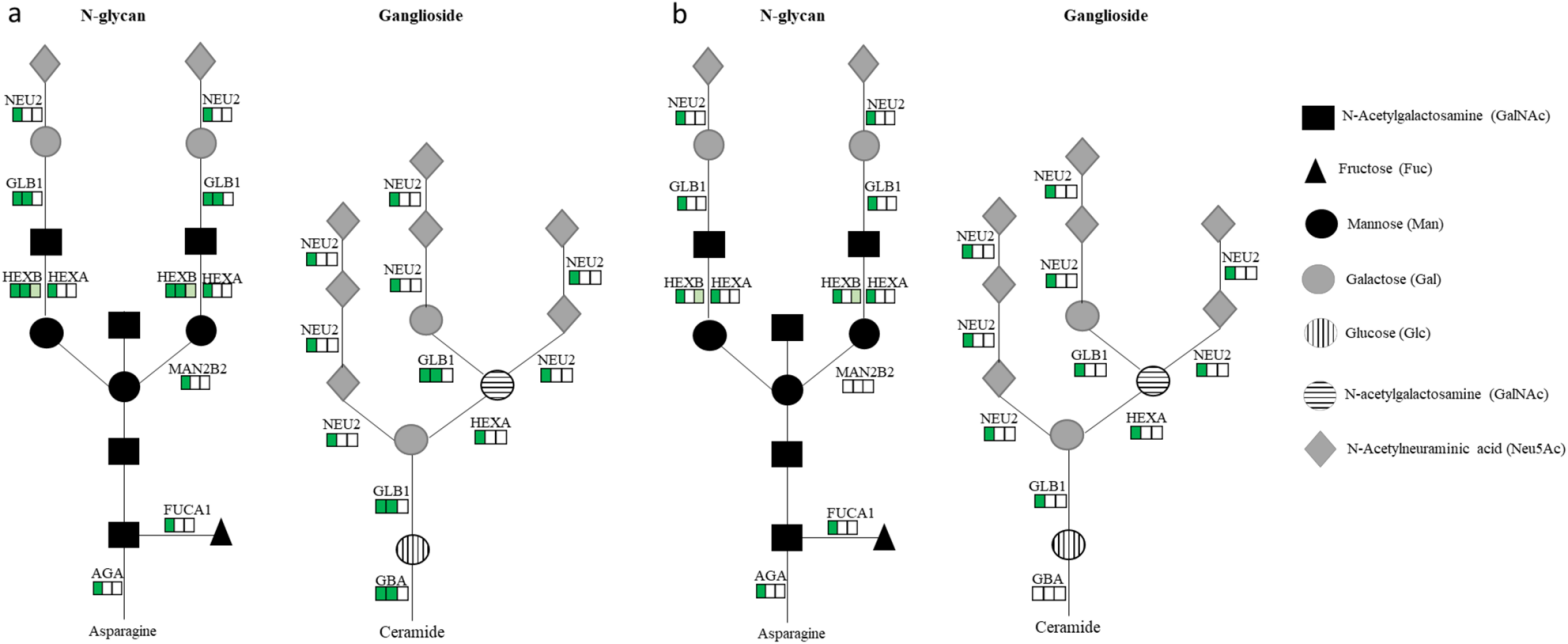
Glycosaminoglycan degradation with the projection of the modulation of the enzymes by ROL (**a**) and RA (**b**). Each square represents the down- (green) or non- modulation (white) of the enzymes at 3 (left square), 6 (middle square) and 12 (right square) months.

A large impact of ROL and RA was observed on glycan metabolism with 14 glycosidases downregulated at 3 months (Fig. 8d). These enzymes are known to be mainly involved in the de-glycosylation of several types of glycans: N-glycans, O-glycans, heparan sulfate, dermatan sulfate, chondroitin sulfate and keratan sulfate (Fig. 9). Comparison of ROL and RA signatures on glycan metabolism revealed some differential impact of the retinoids.

Notably, five enzymes were specifically downregulated by ROL: GAA (acid alpha-glucosidase), GALNS (galactosamine-6-sulfate sulfatase), GBA (glucosidase beta acid), MAN2B2 (mannosidase alpha class 2B member 2) and NAGA (N-acetylgalactosaminidase alpha).

### Long lasting impact of ROL and RA on epidermal barrier-associated proteins

The epidermal barrier-associated proteins related to the eponym function were strongly impacted by ROL and RA application at all time points. This observation suggested a long-lasting effect of the retinoids on these proteins and required further description. 53 DEPs belonged to this superpathway and covered the following functions: ‘cornified envelope components’, ‘Ca^2+^-binding S100 protein family’, ‘intermediate filament’, ‘lipid metabolic process’, ‘serine protease and protease inhibitors’, and ‘desmosome’. Except for desmosome proteins which were all downregulated, and ‘Ca^2+^-binding S100 protein family’ in which all proteins were upregulated, the other functions showed a mixed protein dynamic (Fig. 8e).

A marked dysregulation of numerous keratins was observed after treatment by both retinoids: upregulation of KRT1 (Keratin 1), KRT4 (Keratin 4) and KRT10 (Keratin 10) and downregulation of KRT2 (Keratin 2) and KRT9 (Keratin 9). Some specificity of ROL and RA was also observed with ROL (Keratin 5 (KRT5) and KRT14 (Keratin 14)) or RA (KRT15 (Keratin 15)).

In cornified envelope function, FLG (Filaggrin), IVL (Involucrin), PPL (Periplakin), KRTDAP (keratinocyte differentiation-associated protein), and CALML3 (calmodulin-like protein 3) were upregulated by both retinoids while EVPL (envoplakin) was only upregulated by ROL. Additionally, transglutaminase enzymes TGM1 (transglutaminase 1) and TGM3 were downregulated at all time points, which was confirmed for TGM3 by ELISA (Table 2).

**Table 2:** Evaluation of the impact of ROL and RA application on concentration of TGM3 in the SC as evaluated by ELISA assay. Ratios of the concentration of TGM (versus vehicle) and *P*-value are given for each time point.

TGM3	3 Months		6 Months		12 Months	
	Ratio	*P*-value	Ratio	*P*-value	Ratio	*P*-value
ROL vs vehicle	0.6	0.22	0.87	0.68	0.48	0.072
RA vs vehicle	0.9	0.31	0.71	0.027	0.5	0.025

Proteins associated with lipid metabolism and signaling showed a strong upregulation for FABP5 (FC = 8,8) with ROL and RA, in contrast to a downregulation of eight proteins involved in ceramide synthesis such as ASAH1 (Acid ceramidase) and in cholesterol synthesis as seen for ACAT2 (acetyl-CoA acetyltransferase 2).

## Discussion

This study brings the first temporal in vivo proteomic signatures of ROL (217 proteins) and RA (219 proteins) in the SC with a proteomic analysis strategy based on iTRAQ quantitative proteomic to compare the effect of ROL and RA daily application on skin in a long-term study. The comparison of the two signatures revealed: (1) a high homology between ROL and RA effects (65% of DEPs were in common), (2) the same biological functions impacted and (3) a similar evolution pattern of skin’s molecular response over time which is not surprising knowing that ROL is the metabolic precursor of RA. As it is well known that for comparable concentrations, RA is more effective and more irritating than ROL, we used a much higher concentration of ROL than RA in this study (×12). We analyzed the data as a comprehensive retinoid signature taking into account the substantial difference in concentration. Consequently, any comparison between the 2 actives will be made in the context of this significant difference in concentration. We will first focus on the known and new biological functions impacted by retinoids and then on the temporal aspect of the skin’s molecular response.

Since retinoids are known for their effect on aging signs, we will focus on deciphering the molecular mechanisms leading to anti-aging benefits. One of the best-known hallmarks of aging is the decrease in energy production^17,18^. In our study, energy production was boosted by retinoids^19^ through the upregulation of 5 essential enzymes (ENO1, PGM1 (Phosphoglucomutase 1), PGK1, PKM (Pyruvate kinase M1/2), and GPI) of the glycolysis pathway. This hypothesis can be supported by the finding of Ahmad Alatshan et al.^20^ which showed that RA increases the metabolic pathways towards glycolysis.

Another hallmark of aging is the imbalance of proteostasis^18^, which in skin can be characterized by the balance in proliferation/differentiation of epidermal keratinocytes^21^. An increase in the proliferation of epidermal keratinocytes can be noticed on two levels. Firstly, an increased protein biosynthesis at early time points due to an upregulation of translation initiation factors (EIF6, EIF2S1, EIF4A1), the translation elongation factor (EEF2) and one 60S subunit of ribosome (RPLP2 (60S acidic ribosomal protein P2)). To our knowledge, this is the first report of retinoids’ impact on protein translation which reflects the protein biosynthesis. In addition, this increase in protein production could potentially compensate for the decreased protein and polyamine biosynthesis in aged skin as demonstrated in previous studies^22^. Secondly, there were increased levels of CRAPB2 and FABP5, retinoid binding proteins essential for carrying the RA to their nuclear receptors. In medulloblastoma, it has been shown that the levels of these retinoid binding proteins can lead to either proliferation when FABP5 levels are above CRABP2 levels as shown in our study or to growth inhibition (when CRAPB2 > FABP5)^23,24^. While both proteins were upregulated by the retinoids, FABP5 was found to be at higher increased levels than CRABP2, suggesting an increased proliferation. Furthermore, a positive effect on differentiation was observed at the level of keratin expression, with an upregulation of keratins 1 and 10, both well-known markers of terminal differentiation^25,26^. Additionally, proteins of the epidermal differentiation complex including S100 proteins and FLG were found upregulated after ROL and RA application.

Several studies have also shown that skin aging is accompanied by significant changes in glycosaminoglycans (GAGs)^27^ causing an alteration of their main functions as intercellular communication agents and as regulators of enzymatic activities. This might be linked to the glycan metabolic and catabolic pathways as GLB1 (galactosidase beta 1) is often found to be overexpressed in aged skin^22,28^. In our study, 14 glycosidases involved in the de-glycosylation of several types of glycans were massively downregulated, suggesting the inhibition of shortening of GAGs by both retinoids. In particular, GLB1 and FUCA1 (Alpha-L-Fucosidase 1) were both consistently downregulated by the retinoids. This is to our knowledge the first report of such an important impact of retinoids on the modulation of the glycan degradation pathway. Interestingly, ROL seems to have a higher impact on the glycan degradation pathway as 5 additional glycosidases were regulated by ROL compared to RA in this study. This is probably due to the higher concentration of ROL compared with RA rather than a specific effect of ROL, since the conversion of retinol into retinoic acid is necessary to exert its cutaneous effects. Nevertheless, this interpretation highlights other works such as those by Kafi et al.^29^ showing an increased expression of glycosaminoglycan after ROL treatment that might be through the modulation of enzymes from the glycosidase family and consistent with the parallel work of Donovan et al.^30^ showing that modified glycan structure has been associated with aging.

While the proteomic analysis revealed the impact of ROL and RA on 2 new biological functions, we wanted to determine the impact of long-term topical application of retinoids on skin via the production of temporal in vivo signatures. The temporal in vivo signature obtained for the retinoids revealed the orchestrated molecular events that take place during long term retinoids application. Of the seven superpathways modulated by retinoid application, all functions were enriched at the three time points observed but with less DEPs over time. This observation would suggest that skin reaches an equilibrium after 12 months of treatment.

One of the biological functions for which the impact seems to be long lasting was epidermal barrier-associated proteins. The confrontation of proteomic data and morphological data (in vivo multiphoton microscopy data) shows that, as expected, molecular events precede morphological changes with a greater amplitude of proteins modulations at early times (M3 > M6-M12) whereas epidermal thickening is more significant at late times (M12 > M3-M6). As for the proteomic analysis, morphological modifications are modulated over time mainly with ROL with a significant epidermal thickening at M3 and M12 but not at M6. With RA, epidermal thickening was smaller and later, likely due to the high concentration of ROL used (0.3%), 12 times higher compared with RA concentration (0.025%) in this study^10^.

Some of the orchestrated events that can lead to epidermal thickening reside in the balance of proliferation/differentiation of keratinocytes *i.e.,* epidermal homeostasis. At 3 months of application, the balance in keratins implicated in keratinization was impacted with the up-regulation of KRT1 and KRT10 and down-regulation of KRT2 and KRT9. Keratin balance was modified again at 6 months with the upregulation of epithelial and basal layer keratins (KRT4, KRT5, KRT14). This shift was also supported by the upregulation of proteins precursors of the cornified envelope such as FLG, IVL, EVPL, PPL and KRTDAP, at the early time point of three months. While the impact of retinoids on keratin expression has been previously described^31,32^, the kinetics of the retinoid signature in this study suggests a singularity in the shift between proliferation and differentiation with continuous application of retinoids. In addition, the molecular events leading to the keratinocyte’s proliferation and thus the epidermal thickening requires a stepwise adaptation of the skin that can be detected as early as 3 months after retinoid application.

Similarly, orchestrated molecular events can be observed in protein biosynthesis, with an early upregulation of the initiation of translation (EIF2S1, EIF6 and EIF4A1 were upregulated at 3 months) followed by increased elongation process (EEF2 was upregulated at 6 months by RA and upregulated at 12 months by ROL). Once again, the orchestrated events highlighted the effect on protein biosynthesis and suggested an increased proliferation at early time points.

## Conclusion

The strength of this study is the kinetics over one year, allowing a relevant analysis of the pleiotropic and complex effects of topical retinoids on skin, that are difficult to interpret at a single time.

The molecular signature of ROL and RA was produced using high throughput proteomics technology. To our knowledge, this is the most exhaustive proteomic signature produced to date revealing the effects of ROL and RA on skin’s biological functions. In addition, we have shown that a simple non-invasive skin surface sample associated with a proteomic analysis enables to monitor the response to retinoids over time, opening the door to more personalized anti-aging treatment.

This study leads to highlight little or undescribed functions, in the mechanisms of action of ROL and RA including new function such as glycan shortening and proteins translation/biosynthesis. To go further, complementary in vitro or in vivo studies such as glycomics, metabolomics and protein turnover would be helpful to validate our new hypotheses. Furthermore, this new ROL signature could open the way to the selection of more powerful and better tolerated new anti-aging products.

## Supplementary Information

Supplementary Information.

## Data Availability

All data generated or analyzed during this study are included in this published article (and its supplementary information files).
